# Psychological Prehabilitation for People Undergoing Autologous Stem Cell Transplant: A Qualitative Study

**DOI:** 10.1002/pon.70454

**Published:** 2026-04-15

**Authors:** K. Wilkin, F. Lynch, J. Todd, V. White

**Affiliations:** ^1^ School of Psychology Deakin University Victoria Australia; ^2^ Psychology Department Barwon Health Victoria Australia

## Abstract

**Background:**

Autologous stem cell transplant (AuSCT) improves survival in haematological cancers, yet often involves a lengthy recovery associated with a myriad of psychological and physical challenges. Psychological prehabilitation is recommended to improve functional outcomes by addressing psychological concerns and promoting optimal mental and physical health prior to AuSCT. Despite this, little is known about the implementation and experiences of psychology‐specific prehabilitation for AuSCT.

**Aims:**

The current study explores the perspectives and experiences of AuSCT recipients and healthcare professionals (HCPs), regarding optimal psychological prehabilitation.

**Methods:**

Qualitative interviews were conducted with AuSCT recipients and HCPs experienced in working with AuSCT recipients. AuSCT recipients were recruited at a regional Victorian public health service offering psychological prehabilitation. Purposive sampling was used for HCPs, ensuring a range of professions participated. Reflexive thematic analysis was conducted on interview data, with triangulation utilised to identify themes.

**Results:**

Nine AuSCT recipients and 12 HCPs participated. All themes were shared across AuSCT recipients and HCPs. Four themes (each with subthemes) were identified: Fostering Psychological Preparedness for Transplant *(Exploring Expectations, Addressing Psychological Concerns),* Getting Through the Long Haul *(Prioritising Social* *Support, Planning Emotional and Practical Care),* Strengthening Engagement with Psychology *(Destigmatising Psychology, Psychology Enhances Overall Prehabilitation)* and Looking Toward Sustainability *(Individualised Stepped‐Care, Screening and Risks)*.

**Conclusions:**

Psychological prehabilitation is considered crucial for optimal preparation and recovery from AuSCT, however stigma and limited psychology resources can impact access. Future research should investigate sustainable models of psychological prehabilitation, such as stepped care, to adequately meet the needs of this growing population.

## 1 Introduction

Although Stem Cell Transplants (SCT) for haematological cancers offer the potential for cure or longer remission periods, they are a high risk procedure that can have long lasting negative impacts on quality of life (QoL) and psychological wellbeing [1]. Autologous SCT (AuSCT) involves the harvesting and returning (transplantation) of an individual's own haematopoietic stem cells, to replenish bone marrow stores depleted through intensive chemotherapy or radiotherapy treatment [2]. Approximately 1400 Australians underwent AuSCT in 2024 [3], an increase of 25% since 2015, due to advances enabling AuSCT use for a broader range of cancers and in populations previously considered too high‐risk [4, 5]. Multi‐disciplinary teams (MDT) are increasingly interested in how to best meet the physical and psychological needs of the growing number of individuals undergoing AuSCT (referred to throughout as AuSCT recipients) [6].

AuSCT commonly requires an isolating hospital stay and recovery, with return to pre‐morbid function often taking several months to years [7]. Infectious and non‐infectious complications in the three months following AuSCT are relatively common, with a longitudinal study involving 3552 patients finding 46% had at least one complication over this time period [8]. AuSCT recipients have reported feeling unprepared for the physical impacts of the treatment and required recovery period [9, 10]. Other work has shown health‐related QoL declines rapidly from pre‐transplant until 10–14 days following AuSCT, with return to baseline levels taking three months [11] or longer [8].

AuSCT has also been associated with poor psychological outcomes including symptoms of depression and anxiety [4, 12]. While recent research into AuSCT‐specific psychological outcomes is lacking, a 2009 prospective cohort study from the United States (*n* = 94) found the prevalence of clinical levels of anxiety and depression increased pre‐transplant (39% and 40% respectively) to post‐transplant (45% and 48% respectively) [13]. A recent feasibility randomised controlled trial (RCT) found 42% of participants (89% AuSCT) experienced clinical levels of distress during the acute transplant phase [14].

Pre‐existing psychological distress may increase the likelihood of complications following AuSCT, including self‐reported cognitive changes, slower white blood cell recovery and decreased overall survival [15, 16]. One study following individuals over the first 100 days post‐AuSCT, found those with clinical levels of depression pre‐AuSCT had fewer days alive and out of hospital compared to individuals without depression [17]. Another study using hospital admission data to assess impacts of psychological risk factors on in‐hospital mortality in the 30 days following SCT, found risk of mortality significantly increased for those with depressive disorders, anxiety disorders and post‐traumatic stress disorder [18]. Addressing an individual's psychological functioning prior to and following AuSCT is imperative to optimise physical and mental health outcomes [4, 6, 19].

Evidence suggests that multidisciplinary prehabilitation programs prior to intensive cancer treatment can improve functional outcomes, fatigue, anxiety symptoms and emotional well‐being [20, 21, 22, 23]. However, despite recommendations to include psychology in multidisciplinary programs [24], much of the literature focuses on physiotherapy and nutrition intervention [6, 23, 25, 26, 27, 28, 29]. Multiple barriers have been identified to translating formalised multidisciplinary AuSCT prehabilitation programs that include psychology into healthcare environments including: time poor clinicians, limited resources, disease and appointment burden, and access [14, 22, 30].

Given the limited evidence regarding optimal psychological prehabilitation for AuSCT, further exploration is needed to understand the psychological needs of AuSCT recipients and potential of psychological prehabilitation in meeting these needs. To this end, this study used a qualitative design to understand the perspectives and experiences of Australian AuSCT recipients and HCPs regarding psychological prehabilitation. To date, limited voice has been given to AuSCT recipients and HCPs involved in their care, regarding optimal psychological prehabilitation programs. Understanding the experiences of both AuSCT recipients and HCPs can inform models of care helping to improve uptake and sustainability [14, 22, 30].

## 2 Methods

### 2.1 Design and Procedure

A qualitative design, involving one‐on‐one interviews with AuSCT recipients and HCPs. Ethics approval was obtained from Barwon Health Human Research Ethics Committee (HREC) (Reference: 23.133) Reporting follows the consolidated criteria for reporting qualitative studies (COREQ) [31].

### 2.2 Participants

#### 2.2.1 AuSCT Recipients

AuSCT recipients were recruited from a regional public health service in Victoria, Australia that offers AuSCT in the treatment of haematological cancers (approximately 30 per year). Prehabilitation including dietetics, physiotherapy and psychology is currently offered to all AuSCT recipients. AuSCT recipients were eligible if they: had undergone AuSCT in the previous 2 years, were aged ≥ 18 years, could read and speak English and were seen by clinical psychology before, during or after AuSCT. Those undergoing tandem transplants (a second planned transplant three to 6 months after the first) were eligible. AuSCT recipients were ineligible if they were: within 3 months of their AuSCT or experiencing significant symptom burden as indicated by medical records or treating clinician.

Eligible AuSCT recipients were identified by the psychology team and provided with study information by their treating clinicians during outpatient appointments. Study information sheets outlined study methods, reason for study and study team. Consent to contact from the research team to provide more information about the study was sought at this time. Invitation letters were also sent to eligible patients by the psychology department that included study information and a Quick Response (QR) code to indicate consent to be contacted. In addition, study flyers that included the QR code were available in outpatient waiting areas. Consenting participants were telephoned by the research team, provided with additional information about the study if needed, and after reconfirming consent, scheduled interviews.

#### 2.2.2 HCPs

HCPs involved in the care of AuSCT recipients were eligible if they worked in a tertiary public hospital and could read and speak English. There were no exclusion criteria. A purposive sample was used, with specific types of health professionals (e.g., psychologist, nurse, doctor) identified for inclusion. HCP contact details were obtained from information held by researchers or from publicly available information on hospital websites. Identified HCPs were sent an invitation email including detailed study information, and a link to provide consent and schedule an interview.

### 2.3 Interviews

Interviews were conducted online via Zoom between December 2023 and April 2024. Consent to participate, and record and transcribe the interview was reconfirmed before the interview commenced. Transcripts were not returned to participants for review.

Interviews were semi‐structured and focused on: perceived outcomes of interaction with psychology, barriers and enablers to accessing psychological support before AuSCT, mental health resources provided and recommended psychological assessment or interventions (see Table S1 for interview guides). Demographic information was obtained at the beginning of interviews. Interviews were conducted by K.W., a Master of Psychology (Clinical) student. Average interview length was 26.29 min (20.46–42.29, SD = 6.05) for HCPs and 38.19 min (19.44–53.39, SD = 11.15) for AuSCT recipients.

### 2.4 Analysis

Transcribed interview data was analysed using reflexive thematic analysis, using NVivo 14 [32]. AuSCT recipient and HCP data were analysed separately, using triangulation to identify themes [33]. Analysis was guided by a phenomenological framework; themes were interpreted from participant reflections using an inductive approach [34]. Data analysis was led by K.W., with regular input and review by V.W. and S.F. Codes, themes and subthemes were identified, discussed and agreed on by the wider research team. Continual collaborative discussion allowed refinement of codes and themes, and discussion of any biases and assumptions that might be influencing understandings and interpretation of interview data [35]. Quotes supporting themes and subthemes from AuSCT recipients and HCPs are provided. The all‐female research team had backgrounds in clinical psychology (F.L.), neuropsychology (J.T.), psycho‐oncology research (V.W.; F.L.) and oncology nursing (K.W.). A reflexivity diary was maintained by K.W. following each interview.

Reflexive thematic analysis emphasises interpretive richness of interview data [36]. Our targeted sample of AuSCT recipients and HCPs provided sufficient depth to support a coherent thematic interpretation. Additional data collection was considered unlikely to add new or greater meaning to the analysis.

## 3 Results

The sample consisted of 21 participants: nine AuSCT recipients and 12 HCPs. Participant demographics are presented in Table 1. Although numbers interviewed for both groups were limited, data sufficiency was reached with information from the latter interviews confirming earlier interview results [34].

**TABLE 1:** Demographic characteristics of AuSCT recipients and health care professionals participating in interviews.

AuSCT recipient characteristics	*N* = 9
Gender (*n*)	
Male	6
Female	3
Other	0
Age in years (*M*, SD)	66.7 (3.6)
Range (years)	59–71
Months since current diagnosis (*M*, SD)	13.2 (3.0)
Months since last AuSCT (*M*, SD)	9.1 (3.2)
Tandem^a^ AuSCT (*n*)	2
Received psychological prehabilitation (*n*)	9
Diagnosis	
Myeloma (multiple or plasma cell) (*n*)	6
Diffuse large B‐cell lymphoma (*n*)	3

Health care professional characteristics	*N* = 12
Years Working with AuSCT recipients (*M*, SD)	9.3 (5.7)
Range	1.5–19
Location	
Metropolitan	4
Regional	8
Discipline	
Clinical nurse specialists (CNS)	4
Outpatient CNS	3
Inpatient CNS	1
Clinical psychology	4
Clinical haematologist	1
Other allied health (DT^b^, PT^c^)	3

^a^ Tandem = two planned AuSCTs within a 3–6 month period.

^b^ DT = dietician.

^c^ PT = physiotherapist.

### 3.1 AuSCT Recipients

Forty‐eight individuals referred for psychological prehabilitation for AuSCT were screened for eligibility and 18 identified as potentially eligible, of whom nine (50%) consented to participate. All consenting AuSCT recipients had participated in psychological prehabilitation. Response rates were highest for those invited during follow‐up appointments (5/10, 50%) compared to letters (3/8, 38%); one participant was recruited via the flyer.

### 3.2 HCPs

A total of 25 HCPs were invited, and 12 agreed to participate (54%). A range of disciplines were represented including nursing, haematology, clinical psychology, dietetics and physiotherapy (see Table 1).

### 3.3 Qualitative Themes

Four main themes were identified: *Fostering Psychological Preparedness for Transplant*, *Getting Through the Long Haul*, *Strengthening Engagement with Psychology* and *Looking Toward Sustainability*. All themes and their subthemes were common to both AuSCT recipients and HCPs. Themes, subthemes are summarised in Table 2 with Table 3 showing supporting quotes.

**TABLE 2:** Summary of themes and subthemes developed through thematic analysis.

Themes	Subthemes
Fostering psychological preparedness for transplant	• Exploring expectations • Addressing psychological concerns
Getting through the long haul	• Prioritising social support • Planning emotional and practical care
Strengthening engagement with psychology	• Destigmatising psychology • Psychology enhances overall prehabilitation
Looking toward sustainability	• Individualised stepped‐care • Screening and risks

**TABLE 3:** Themes and subthemes with illustrative quotes

	Theme	Sub‐themes	Illustrative quotes	
			Health care professionals	AuSCT recipients
1	Fostering psychological preparedness for transplant	Exploring expectations	‘…Often they will kind of say, “oh, you told me I was going to be feeling like this and, you know, I didn't really believe you. But now I kinda get what you said’. (outpatient CNS, HCP 2) ‘…There seems to be a difference (in adjustment) in people that seemingly have the same drop in function objectively… it's also how they perceive the change that's happening and what it means and how equipped they are to cope with it…”’ (clinical psychologist, HCP 9)	‘They talk to you about it, but living through it is different…you've given it to me on paper, but what's my reality in the middle here?.. That void was what I needed a bit of help with a gap in the middle… just having some strategies to manage that is important….that's what I found the hardest… that's where some psychological support would be really valuable….It was for me.’ (AuSCT recipient 2) ‘…A diagnosis alone is life changing…. You start to think’, oh, am I gonna die?’…that's a really crucial time that a patient could use some psychological help’. (AuSCT recipient 9) ‘I had a breakdown (following diagnosis)….I started to cry and breakdown, cause I had no idea what I was in store for. Not an iota of a clue’. (AuSCT recipient 1)
		Addressing psychological concerns	‘I would say, yeah, grief, anger, sadness, being overwhelmed are probably the biggest things…It's just so much to get the head around… the new diagnosis, the new treatment, plus the transplant…’ (HCP 5 (cancer nurse specialist)	‘I remember just some techniques about grounding yourself, how I breathe, how I'd sit, how I coped with things…’ (AuSCT recipient 2) ‘…don't sit there waiting to feel better so you can go do something…. Go and do something and then you'll feel better….it's back to front…it almost works the opposite way’. (AuSCT recipient 4) ‘And the closer I got, the more anxious I got and that's what it was. I just… I thought I was having a breakdown or something because I just didn't know what was going on with me… I'm relatively a strong person and I've never experienced anything like that’. (AuSCT recipient 8) ‘…I think too that (it's important) you sort of talk [to] somebody who's obviously educated in that field… they just know what you're going through…they see you actually as a human being’. (AuSCT recipient 3)
2	Getting through the long haul	Prioritising social support	‘I might be thinking about…if you can't get outside that day, and you know that's really confronting for them because they have to think, “oh, no, I do everything myself” and I'm like, OK, who would you call if you could? ….you're not gonna be able to do any gardening whilst you don't have an immune system…. Who would you call?”’ (clinical psychology registrar, HCP 7) ‘So for me I just go immediately to them (Myeloma Australia) and know that they will support my patients ongoing.…I think having the support network involved and knowledgeable as well helps massively.’ (outpatient CNS, HCP 5)	‘…(Psychologist) suggested having a couple of people to check in with you when you're in a hospital…key people that you could trust that you could really express how you're feeling…And so I chose two people and that's worked really, really well for me’. (AuSCT recipient 2) ‘it's better to have somebody in the background to organise all the visits and all that sort of stuff. So, you know, it's not that overwhelming’. (AuSCT recipient 5)
		Practical and emotional care	‘…The ones that come in and have all their family photos ready…. They get care packages and phone calls from family… I just think those patients do so much better….’ (Inpatient CNS, HCP 4)	‘… (my wife) went through the whole process with me… there's a lot you don't take in… it helps to have another set of ears’ (AuSCT recipient 6) ‘I had these sessions where you could sit around with a group like, like people that have got cancer….And suddenly you think well, you know, it's not about little old me at all. You know, there's a vast number of people going through something very similar.’ (AuSCT recipient 3)
3	Strengthening engagement with psychology	Destigmatising psychology	‘…They might not always know what the psychologist does in that context… they'll think “I don't need the psychologist. I'm fine…”’ (Dietician, HCP 12) ‘I think they think…if they're offered psychology, it's a sign that they're not coping and… they're gonna be labelled with depression, anxiety …given a diagnosis that they don't want in addition to everything else that is going on’ (physiotherapist, HCP 10) ‘… the most important thing is about it being integrated as routine care, and it being presented that way to patients so… “all of our patients will be referred or asked about a psychologist. This does not mean that you're not coping… it is about how we prepare you psychologically for treatment” so that it's not kind of pathologising…’ (clinical psychologist, HCP 6)	‘I sort of was apprehensive about talking to a psychologist… I thought it was more like a psychiatrist…. They (psychologists) don't diagnose, whereas the psychiatrist does… as I said psychology, they just ask you questions….it just helps (to) talk to someone’ (AuSCT recipient 8) ‘I've generally found counsellors (previously)…. really have not been a lot of value… (Following psychological prehabilitation) I had opened my eyes to what psychology was…I really didn't know. And I've got a lot of time for that (psychological prehabilitation) counselling process.’ (AuSCT recipient 1)
		Psychology enhances overall prehabilitation	‘I frame psychology as being, you know, a cog in the wheel, and that we need the whole wheel…. I don't sort of differentiate between physical and psychological health.’ (outpatient CNS, HCP 2) ‘We're here to support you to prepare most effectively psychologically and also, support your ability to engage in the other things that you need to do to be as healthy as possible for transplant.’ (clinical psychologist, HCP 6) ‘…Targeting (psychology uptake could help) to increase their engagement in prehab as a whole…if we can make it more cohesive in terms of our appointment scheduling… communicate with each other and work together towards these common goals, rather than it being “oh, your physio's done and now you start psychology”’ (physiotherapist, HCP 8)	‘I'd say someone needs to put a helicopter up to see what all these programs are, because they all seem to be working in isolation.’ (AuSCT recipient 1)
4	Looking towards sustainability	Individualised stepped‐care	‘I think there's a balance to be struck there… (which) services probably need to consider… it's probably different for each patient around what's sort of helpful versus overwhelming and motivating versus overwhelming…’ (clinical psychologist, HCP 6) ‘…(We need something) like a birth plan… it might never go as planned and you can't stick to it all the time… let's assume you're gonna feel really, really bad… what would work best for you? Do you want us to call your family, leave you alone, put on some music?’ (haematologist, HCP 11) ‘I think we need to be creative about how we offer it and how we deliver it so that we are providing the best psychological support… it's tricky because I think each individual patient is different in terms of what's going to be best for their well‐being….’ (physiotherapist, HCP 10) ‘there's not enough psychology funding, and I think that is most places. And then I guess it would only be really the pointy end of the most distressed that we get access to individually. But that may not be what is the most ideal.’ (clinical psychologist, HCP 1) ‘So there's a cancer council form that I tend to give people about dealing with stress… There's a whole range of booklets that…. refer to mental health.’ (outpatient CNS, HCP 3)	‘I didn't really need it…I just knew it was just part of the process i needed to go through to…to stay alive, basically.’ (AuSCT recipient 5) ‘It's (AuSCT) not that hard of a thing to go through (mentally) … but it does do a lot to your body.’ (AuSCT recipient 6)
		Screening and risks	‘…Otherwise you're just sitting there going. “Oh, I'm just going on my gut feel here and I don't have any solid evidence to say that this might be a step too far for them.”’ (outpatient CNS, HCP3) ‘It's quite crucial that we're at least doing some sort of screen to understand where someone is sitting. I Think that it's very realistic and expected that people would experience some level of distress … I would want to be able to see whether that is elevated in any way…’ (clinical psychologist, HCP 9) ‘…(The) goal in having patients have a psychological assessment and support through the process is to try and pick… those who won't cope very well … and whether or not there's any way to pre‐empt that. But the people who are probably psychologically unfit to undergo a transplant are pretty obvious even to us non‐psychologists. It's the ones…where we are blindsided that we would, I guess like some help predicting.’ (haematologist, HCP 11)	‘I suppose if you've got people that already are on the edge and they're going through all this, no matter what sort of cancer it is they would definitely need people to talk to, I think.’ (AuSCT recipient 8) ‘(Younger people) would struggle to work and raise kids and deal with this at the same time, that's for sure.’ (AuSCT recipient 7)

#### 3.3.1 Theme One: Fostering Psychological Preparedness for Transplant

AuSCT recipients and HCPs highlighted several areas of focus for psychological prehabilitation, which they believed could optimise AuSCT preparation.

##### 3.3.1.1 Exploring Expectations

AuSCT recipients and HCPs reported a discrepancy between information provided and absorbed pre‐transplant and thought psychology offered a way to reduce this gap. Many AuSCT recipients expressed feeling both overwhelmed with the amount of information received pre‐transplant and surprised by the effects of transplant, especially the reduction in QoL during recovery. AuSCT recipients and HCPs believed recipients' focus on survival and a ‘whirlwind’ schedule pre‐transplant diminished their ability to consider longer‐term impacts. Both groups suggested psychology was uniquely placed to help AuSCT recipients explore recovery expectations and provide strategies for managing difficulties arising during AuSCT.

##### 3.3.1.2 Addressing Psychological Concerns

Both groups described unique psychological challenges to be addressed that could be experienced before, during and after AuSCT including: anxiety, overwhelm, fear, grief, uncertainty, low mood and adjustment difficulties. There were conflicting views among AuSCT recipients regarding the benefits of a ‘brave’ mindset which some thought was protective, while others believed this approach contributed to avoidance of difficult emotions and eventual overwhelm. AuSCT recipients discussed how the non‐judgemental person‐centred support offered by psychologists with experience in AuSCT care helped them to discuss difficult emotions, learn stress management skills, and validate and accept their experience.

In addition to the focus on emotional support and strategies, AuSCT recipients and HCPs discussed other areas psychological prehabilitation could address. For AuSCT recipients, this focused on emphasising healthy lifestyle behaviours and boosting activity to improve overall wellbeing during recovery. Psychologists discussed different therapeutic approaches they use to address specific issues including Cognitive Behaviour Therapy (CBT) (particularly behavioural activation, decision making) to manage depressive and anxiety symptoms, Acceptance and Commitment Therapy (ACT), motivational interviewing, stress management, relaxation and mindfulness. However, they were cautious regarding the use of cognitive reframing given the uncertainty involved in the procedure, preferring a focus on emotional processing.

#### 3.3.2 Theme Two: Getting Through the Long Haul

AuSCT recipients and HCPs recognised that recovery from AuSCT is long and isolating, recommending psychological prehabilitation includes planning of support from support networks that meets a wide range of practical and emotional needs to improve outcomes. Two subthemes were identified: prioritising social support and planning emotional and practical care.

##### 3.3.2.1 Prioritising Social Support

Mobilising social networks prior to AuSCT to assist throughout the ‘long haul’ recovery associated with AuSCT, was perceived by AuSCT recipients and HCPs as crucial to establish sustainable support that endures beyond the healthcare system and enhances both physical and emotional outcomes. AuSCT recipients and HCPs believed psychological prehabilitation provided an opportunity to *proactively* identify and mobilise a range of social supports to meet a variety of potential needs throughout an individual's transplant experience.

Post‐transplant peer support was consistently endorsed by AuSCT recipients as useful to help validate their experience and reduce isolation during recovery. However, there was no consensus regarding peer support pre‐transplant, with some participants thinking this might worsen their already heightened emotional state, while others thought hearing about positive experiences might be useful.

##### 3.3.2.2 Planning Emotional and Practical Care

Both AuSCT recipients and HCPs recognised the period following transplant as socially isolating due to the multiple factors impacting engagement with usual activities including: lowered immunity, reduced function, fatigue, multiple appointments and being unable to travel. AuSCT recipients reported their support network was crucial to getting through this period by assisting them with information gathering, planning, activities of daily living and emotional support. HCPs believed those with regular, pre‐planned, practical input from family and friends coped better with the ‘long haul’ following AuSCT.

#### 3.3.3 Theme Three: Strengthening Engagement With Psychology

AuSCT recipients and HCPs believed that strengthening engagement with psychology pre‐transplant would improve overall outcomes in AuSCT. Both groups discussed barriers to connecting with psychology and suggested that a psychological prehabilitation pathway may overcome these.

##### 3.3.3.1 Destigmatising Psychology

HCPs observed that stigma around psychology reduced referral acceptance from AuSCT recipients, particularly for older individuals. AuSCT recipients' misunderstandings of why they were referred to psychology contributed to this stigma.

Most AuSCT recipients reported they did not know or were too overwhelmed to consider what psychological prehabilitation might offer, with some AuSCT recipients seeing a referral to psychology as indicating their health care team thought they were not coping. Both AuSCT recipients and HCPs discussed how describing psychological prehabilitation as part of routine care could reduce the stigma associated with psychology.

Increasing the visibility of psychology, through for example the provision of short triage phone calls or delivering group psycho‐education sessions, was seen by HCPs as beneficial to improve knowledge of psychology's role within AuSCT and boost participation in psychology programs.

##### 3.3.3.2 Psychology Enhances Overall Prehabilitation

HCPs recognised that psychological prehabilitation optimises *overall* prehabilitation, with many acknowledging that psychological and physical prehabilitation strengthened each other to contribute to improve AuSCT outcomes. For example, some HCPs reported that psychological support for individuals experiencing low motivation pre‐transplant may contribute to improved engagement with physical prehabilitation, optimising the individual's overall preparation.

Improved integration of psychology into the prehabilitation programs was suggested by AuSCT recipients and HCPs as a way to increase uptake of psychological prehabilitation. HCPs recommended a number of ways to facilitate this including: timely referrals, convenient appointment scheduling, creating opportunities for secondary consultations with psychology and regular communication within the team. HCPs thought earlier referrals to psychology might allow psychology to provide input into MDT meetings and the care plan, potentially improving the likelihood that psychology is included in the prehabilitation programs for AuSCT.

#### 3.3.4 Theme Four: Looking Toward Sustainability

HCPs and AuSCT recipients discussed potential strategies to ensure psychological prehabilitation addresses individual needs while remaining sustainable in the context of limited resources. Two subthemes were identified: individualised stepped care and screening and risk (see Table 3 for supportive quotes for each subtheme).

##### 3.3.4.1 Individualised Stepped‐Care

AuSCT recipients and HCPs discussed optimising psychology resources to ensure individuals could receive the level of psychological support needed. Both groups thought it important to develop strategies that could be tailored to the needs of the individual. Within the HCPs, some likened this approach to developing a ‘birthplan’ that could provide coping or management strategies depending on the outcomes experienced. Others suggested the approach would help ensure that patients are not overwhelmed with information they are not yet ready for.

The need to tailor interventions to the individual's needs also emerged from comments from AuSCT recipients, with some noting they did not need the one‐on‐one psychological prehabilitation appointment they attended, while others appreciated all psychology input, starting with the initial triage phone call. Group psychological prehabilitation was mentioned by some AuSCT recipients and HCPs as a potential way to deliver general psychoeducation. However, others were ambivalent about this approach, due to potential distress experienced and hectic schedule pre‐transplant.

Non‐psychology HCPs noted that they provide a number of mental health resources including printed or internet‐based information packs, service specific pre‐recorded videos or referral to external resources. They suggested that in situations where psychology services were limited, using the different disciplines involved in prehabilitation to provide at least some simple psychoeducation information may be an alternative model.

##### 3.3.4.2 Screening and Risks

AuSCT recipients and HCPs discussed potential risk factors for psychological distress before, during or after AuSCT suggesting these could be used to triage psychology referrals. All disciplines were open to using screening tools to assess for psychological risk pre‐transplant and believed this would improve outcomes for AuSCT recipients. HCPs suggested these could be administered around the period of diagnosis, to help identify those most in need of psychological prehabilitation for AuSCT. Non‐psychology HCPs wanted something other than a “gut feeling” to help identify those needing specialised psychological prehabilitation.

Non‐psychology HCPs reported they often used the Distress Thermometer for screening. Psychologists reported using a range of screening tools in an AuSCT context including: Distress Thermometer; Patient Health Questionnaire—4 & 9 item (PHQ), Brief Symptom Inventory (BSI), and Hospital Anxiety and Depression Scale (HADS).

Risk factors for psychological distress throughout the transplant process identified by AuSCT recipients and HCPs included: younger age, having dependants, social and geographical isolation, pre‐existing mental health diagnoses and financial strain.

For HCPs, additional risk factors included: substance use, high emotional preoccupation (i.e., focusing on emotional consequences of AuSCT), low motivation, male gender, suicidal ideation and cultural diversity. HCPs also believed older individuals may be at risk due to hesitation in engaging with mental health supports generally. Psychologists reported pre‐existing mental health diagnoses could be a risk or protective factor, attributing the latter to being attuned to early warning signs and coping strategies for distress.

## 4 Discussion

The current study aimed to provide insight into optimal psychological prehabilitation for AuSCT from the perspectives of AuSCT recipients and HCPs working in this field. Findings revealed four main themes shared by AuSCT recipients and HCPs: *Fostering Psychological Preparedness for Transplant, Getting Through the Long Haul, Strengthening Engagement with Psychology, Looking Toward Sustainability.* Themes and subthemes highlight the potential of psychological prehabilitation at the individual and health service level. At the individual level, psychological prehabilitation can optimise psychological preparedness and recovery from AuSCT. At the health service level, reducing stigma and embedding psychology into standard prehabilitation programs may increase engagement with, and visibility of, psychology for staff and patients, thereby reducing barriers to participation and referral [30]. Implementing a stepped‐care model for psychological prehabilitation, could promote consistent referral practices across health professionals [30], supporting long‐term sustainability of programs.

Psychological interventions examined previously have predominantly targeted *rehabilitation* for both AlloSCT and AuSCT populations [37]. The current study is one of the few to focus on understanding perceptions and use of psychological *prehabilitation*, specific to AuSCT. Both AuSCT recipients and HCPs highlighted the potential of psychology in exploring recipients' expectations of AuSCT and helping them to prepare for the ‘long haul’ of recovery. Research has demonstrated that people with cancer recall only up to 60% of cancer or treatment information provided by their medical practitioner, and this recall can be worsened with anxiety [38]. AuSCT recipients identified a difference between their expectations and their experiences of AuSCT, with HCPs also observing this gap. Psychological prehabilitation could potentially bridge this gap through exploration of common emotional reactions to having AuSCT (anxiety, grief, uncertainty, low mood, adjustment) and providing interventions and strategies to help manage these emotions if they arise.

Findings from this study support recommendations that psychology be considered a core element in prehabilitation programs to improve outcomes for AuSCT recipients [26]. However, the availability of psychologists to deliver prehabilitation to all AuSCT recipients is limited given minimal resourcing for psychology in most health services. In line with others [22, 24], participants in the current study recommended incorporating stepped‐care psychological prehabilitation models to adapt to these limitations. These models build on current Australian practices to screen people with cancer for psychological distress, ensuring that all people receive the level of psychological information and support they need [22]. Limited psychology resources contributed to some HCPs in our study suggesting that other disciplines could provide some level of psychological support or resources to AuSCT recipients. Current evidence is mixed on effectiveness of prehabilitation programs involving only physiotherapy and/or dietitians with regards to improving psychological wellbeing or emotional functioning. While some studies suggest improvements in overall QoL [28, 29], little impact has been shown for specific emotional well‐being scales or subscales. However, as emotional wellbeing is generally a secondary outcome, studies conducted to date may be underpowered to detect significant change in these measures.

Previous research has recommended assessing social support prior to transplant and optimising this form of support in recovery [39, 40, 41]. Our findings support this recommendation and suggest the benefits of *actively planning* emotional and practical support during and post‐transplant. Planning for contact from family or social supports during AuSCT recipients' hospitalisation period was seen as helpful in supporting recipients' mental and emotional health during this period. Others have found that the support networks of AuSCT recipients play a pivotal role in providing practical support during and post hospitalisation across a range of areas particularly daily living tasks (e.g., shopping, house and garden maintenance), reducing AuSCT recipients' concerns [39]. Our study found support for planning peer support contact for the period *following* transplant. This approach contrasts with recent research involving transplant recipients (mixed Allo and AuSCT populations), that tested the impact of a 5‐week peer support program that started prior to transplant which was considered acceptable to participants [42]. The different approach to the timing of peer support used in that study to the preferences reported here suggests that more work is needed to understand peer support needs for AuSCT recipients. Our results do suggest that the inclusion of peer support in some capacity may be beneficial post‐transplant.

Although previous research has identified several challenges to implementing psychological prehabilitation for AuSCT in healthcare environments [14, 22], our study revealed stigma relating to psychology as a major barrier. Although several potential paths for addressing stigma were suggested, a key strategy was ensuring psychology prehabilitation was seen as part of usual care. Suggestions for achieving this included upskilling members of the MDT to discuss psychological prehabilitation as routine practice and the broad benefits of psychological prehabilitation. HCPs recommended psychology be further integrated within the MDT to optimise opportunity for input. This approach aligns with previous literature [30], which identified MDT teamwork as an enabler for delivery of prehabilitation for AuSCT. Research is needed to determine if these strategies can address stigma and improve uptake of psychological prehabilitation for AuSCT.

### 4.1 Clinical Implications

To optimise resources and ensure AuSCT recipients are adequately supported, the current study suggests a number of different features could be included in future models of psychological prehabilitation. Potential features include a stepped‐care approach that individualises interventions using comprehensive biopsychosocial screening during the development of medical treatment plans. A focus on boosting social support pre‐transplant may assist AuSCT recipients in enduring the ‘long haul’ of recovery. Normalising psychological support within AuSCT care, and ensuring psychology is considered part of standard care, may reduce stigma and improve uptake of psychological prehabilitation for AuSCT. Potential interventions that may be used at the individual and the health service level to address our four themes are shown in Table 4. Research is needed to determine the utility of these potential interventions to improve the integration of psychology in AuSCT prehabilitation programs, reduce distress, and optimise long‐term QoL for AuSCT recipients.

**TABLE 4:** Possible interventions to address each theme^a^.

Theme	Subtheme	Possible interventions
Fostering psychological preparedness for transplant	Exploring expectations	Psychological intervention options: – Explore procedure and recovery expectations, – Provide strategies for managing difficulties arising during transplant/recovery, – Communication skills training to assist AuSCT recipients to effectively communicate their expectations and ask needed questions of their healthcare team Other: – Psychologists liaison with MDT to inform them of AuSCT recipient's recovery expectations.
	Addressing psychological concerns	Psychological intervention options: – Non‐judgemental person‐centred psychological support – Validation and normalisation – Stress management/emotion regulation skills training – Supporting healthy lifestyle behaviours with education and behaviour change – Behavioural activation – Decision making support – Acceptance and Commitment Therapy – Motivational Interviewing – Relaxation training – Mindfulness – Increasing coping with uncertainty
Getting through the long haul	Prioritising social support	Psychological intervention options: – Identifying social, emotional, and practical support options – Addressing barriers to engaging supports (e.g., shame; communication skills; difficulty problem solving or planning) – Consider linking in with post‐transplant peer support if desired
	Planning emotional and practical care	Psychological intervention options: – Develop task lists where support will be needed – Plan practical support – Plan emotional support to cope with admission (e.g., photos on ward, calls with family)
Strengthening engagement with psychology	Destigmatising psychology	System intervention options: – Normalising psychology when discussing referral – Increase visibility of psychology (e.g., short triage phone calls; delivering group psycho‐education sessions) – Providing information and education on the role of psychology in AuSCT prehabilitation – Integrating psychology into the MDT
	Psychology enhances overall prehabilitation	System intervention options to enhance these effects: – Integration into MDT and prehabilitation programs – Early referral to psychology to inform MDT plans – Convenient appointment scheduling close to other MDT appointments – Create opportunities for secondary consultations with psychology – Psychology attending MDT meetings and regular communication with the team
Looking toward sustainability	Individualised stepped‐care	System intervention options: – Screen for level of psychological intervention required – Provide low intensity interventions to those with low needs (e.g., psychoeducation resources, mental health resources, internet‐based information packs, pre‐recorded videos) – Provide high intensity interventions to those with high needs (e.g., 1:1 therapy) – Consider group therapy as an option, though delivery difficulties due to scheduling and potential distress need to be addressed
	Screening and risks	Screening intervention options: – Could be conducted by referring clinicians prior to referral, a prehabilitation clinician or allied health assistant, or psychology following referral prior to being allocated to an intervention – Use of validated screening tools (e.g., distress thermometer, PHQ‐4, PHQ‐9, GAD‐7, HADS) – Identify potential socio‐demographic risk factors (e.g., age, gender, carer status, isolation, financial strain, language, etc) – Clinical interview to screen for potential psychological risk factors (pre‐existing mental health concerns, substance use, high emotional preoccupation with consequences of AuSCT, low motivation, suicidal ideation)

^a^ Interventions may not yet be trialled and determined effective. It is recommended that future research further explore the appropriateness and effectiveness of these suggested intervention possibilities.

### 4.2 Study Strengths and Limitations

A strength of the current study was the specific focus on psychological prehabilitation for AuSCT, an area which has received little attention. Triangulation of perspectives and experiences of AuSCT recipients and HCPs facilitated a multifaceted understanding of optimal psychological prehabilitation for AuSCT. A broad range of expertise was captured through HCP interviews, given this group represented a range of disciplines and geographical locations, with most HCPs reporting many years of experience. Capturing perspectives of both AuSCT recipients and HCPs facilitated exploration of how psychological prehabilitation models for AuSCT might be tailored to real world settings, addressing a gap in existing literature [14, 30]. While the number of interviews conducted with AuSCT recipients and HCPs was relatively small, interviews provided rich data that enabled answers to our research questions through our reflexive thematic analysis [36]. Confirming themes across the two participant groups supports the veracity of our findings.

AuSCT recipients were recruited from one public health service, with perspectives of those who had not received psychological support as part of their AuSCT care lacking from this study. Furthermore, AuSCT recipients recruited were aged 59 years and older, suggesting a gap in findings regarding the needs of younger individuals who may be more likely to be working and have dependants. Future research should explore perspectives of the above groups. Work exploring the impact of intent for transplant, disease status and ongoing treatment on experiences of AuSCT is also needed to build an understanding of potential of psychological prehabilitation for all AuSCT recipients.

## 5 Conclusion

AuSCT is a stressful procedure, involving a lengthy recovery which is associated with a myriad of psychological and physical challenges for recipients. Psychological prehabilitation tailored to the individual and integrated within the MDT is essential to help people manage the unique psychosocial challenges they may face during this period. Findings from this study highlight the need for future research to develop a sustainable model of psychological prehabilitation that can assist people to manage the emotional impact of AuSCT before, during and after this procedure. A stepped‐care approach that allows all AuSCT recipients to receive the level of psychological support they need may be useful, however further research is needed to demonstrate feasibility and utility of this model.

## Author Contributions

Conceptualisation: F.L., K.W., J.T., V.W. Interview construction: F.L., K.W., J.T., V.W. Analysis: K.W. Results interpretation: F.L., K.W., V.W. Writing – original draft: K.W. Writing – review and editing: F.L., K.W., J.T., V.W. All authors have read and agreed to the published version of the manuscript.

## Funding

This work was conducted as part of the requirements for the award of Master of Psychology (Clinical) undertaken by Ms Kathryn Wilkin. There was no funding to declare for this project.

## Ethics Statement

The study's procedures were approved by Barwon Health's Human Ethics Advisory Group (Reference: 23.133).

## Consent

Informed consent was obtained from all participants prior to their interview.

## Conflicts of Interest

The authors declare no conflicts of interest.

## Supporting information


**Table S1:** Interview guides for patients and health care professionals.

## Data Availability

The data that support the findings of this study are available on request from the corresponding author. The data are not publicly available due to privacy or ethical restrictions.
