# Supplementary material for: Psychological Prehabilitation for People Undergoing Autologous Stem Cell Transplant: A Qualitative Study
Source: Psychooncology. 2026 Apr 15;35(4):e70454. doi: 10.1002/pon.70454 (PMC13084209; doi:10.1002/pon.70454)
Supplement: Supplementary file 1 — Table S1: Interview guides for patients and health care professionals. [file PON-35-e70454-s001.docx]

**Supplementary Table 1: Interview guides for patients and Health Care professionals**

**Patient interview guide.**

- How you were feeling around the time of your stem cell transplant? (*Prompts:* What about before? During? After?)
- Could you talk about your experiences and how the transplant may have impacted your mental health or wellbeing in any way
- What were your biggest challenges before, during, and after the transplant?
- Tell me about your experience of the psychology support you received before, or after your transplant. What did you find helpful? What was least helpful?
- What could have improved these sessions? Was there anything that you thought of afterwards that may have been useful?
- Do you feel your engagement with psychology helped to prepare you for your stem cell transplant or recovery? If so, what were the key factors?
- When is the best time for people to have psychology support around their transplant? Why?
- What support do you think people need before their transplant to help their wellbeing?
- What would make it easier or help people to engage with psychology before their stem cell transplant?
- What information would be helpful for you before your transplant? What sort of timing would be optimal?
- Are there particular groups or situations where you feel psychology support before a transplant is most needed?
- What psychology support do you think is needed *after* stem cell transplant?
- What do you think are the key factors that helped improve or maintain your mental health or wellbeing throughout stem cell transplant?
- What is your preferred way to receive psychology support??
- Anything else you would like to add regarding psychological preparation for stem cell transplant?

**Health Care Professionals**

What are the most significant mental health and wellbeing concerns for patients before transplant? During transplant? And after transplant?

**Questions for medical professionals and allied health (non-mental health clinicians):**

- What is your understanding of the psychological services or support available to patients undergoing autologous stem cell transplant within your organisation?
- Do you find patients ask questions relating to their psychological health during consultations with you (either before or after transplant)?
- What do you most need to know to best support patients’ psychological health before or after their transplant?
- Who most needs psychological support before transplant? What about after transplant? Who needs support?
- When do you think patients should be offered psychological support in relation to their transplant?
- What do you think are the benefits of psychology support for patients and for their carers before transplant?
- What do you think are the key factors that help improve/maintain psychological wellbeing before, during and following stem cell transplant?
- What would make psychology services easily accessible to stem cell transplant patients within your organisation?

**For mental health clinicians only:**

- What makes psychology services accessible for people undergoing stem cell transplants?
- What sort of psychological support do you believe to be most effective/important for a patient during their prehabilitation for stem cell transplant?
- What aspects of the psychological support prior to stem cell transplant do you feel could be improved and why?
- Are there particular points along a patient’s stem cell transplant journey that you feel it crucial to provide psychological support and why?
- What do patients and healthcare professionals need to know about psychological prehabilitation for those undergoing stem cell transplant?
- What helps or hinders your ability to provide psychological support to individuals prior to undergoing stem cell transplant? What about after stem cell transplant? (e.g., telehealth, appointment flexibility, illness etc)
- What format do you think is best for psychological support before or after transplant? (e.g., 1:1 sessions; group sessions; information; telehealth; phone calls face-to-face)
- Who most needs psychological support before or after transplant? Do you recommend any screening measures to determine this?
- Do you provide patients with information or external resources for them to access further information about their mental health prior to stem cell transplant? What does this entail?
